# Conceptualizing outcomes for use with the Consolidated Framework for Implementation Research (CFIR): the CFIR Outcomes Addendum

**DOI:** 10.1186/s13012-021-01181-5

**Published:** 2022-01-22

**Authors:** Laura J. Damschroder, Caitlin M. Reardon, Marilla A. Opra Widerquist, Julie Lowery

**Affiliations:** VA Center for Clinical Management Research, VA Ann Arbor Healthcare System, 2215 Fuller Road, Ann Arbor, MI 48105 USA

**Keywords:** Implementation science, Antecedent assessments, Implementation outcomes, Anticipated outcomes, Actual outcomes, Innovation outcomes, Implementation framework, Evaluation methods, Theory, Consolidated Framework for Implementation Research

## Abstract

**Background:**

The challenges of implementing evidence-based innovations (EBIs) are widely recognized among practitioners and researchers. Context, broadly defined as everything outside the EBI, includes the dynamic and diverse array of forces working for or against implementation efforts. The Consolidated Framework for Implementation Research (CFIR) is one of the most widely used frameworks to guide assessment of contextual determinants of implementation. The original 2009 article invited critique in recognition for the need for the framework to evolve. As implementation science has matured, gaps in the CFIR have been identified and updates are needed. Our team is developing the CFIR 2.0 based on a literature review and follow-up survey with authors. We propose an Outcomes Addendum to the CFIR to address recommendations from these sources to include outcomes in the framework.

**Main text:**

We conducted a literature review and surveyed corresponding authors of included articles to identify recommendations for the CFIR. There were recommendations to add both *implementation* and *innovation* outcomes from these sources. Based on these recommendations, we make conceptual distinctions between (1) anticipated implementation outcomes and actual implementation outcomes, (2) implementation outcomes and innovation outcomes, and (3) CFIR-based implementation determinants and innovation determinants.

**Conclusion:**

An Outcomes Addendum to the CFIR is proposed. Our goal is to offer clear conceptual distinctions between types of outcomes for use with the CFIR, and perhaps other determinant implementation frameworks as well. These distinctions can help bring clarity as researchers consider which outcomes are most appropriate to evaluate in their research. We hope that sharing this in advance will generate feedback and debate about the merits of our proposed addendum.

Contributions to the literature The CFIR Outcomes Addendum:

- Conceptualizes types of outcomes for use with the Consolidated Framework for Implementation Research (CFIR), one of the most widely used implementation science frameworks.
- Clarifies conceptual distinctions between (1) anticipated implementation outcomes versus actual implementation outcomes, (2) implementation outcomes versus innovation outcomes, and (3) CFIR-based implementation determinants versus innovation determinants.
- Guides researchers to choose and describe which types of outcomes their studies are proposing to address, carefully consider the determinants that can affect those outcomes, and in turn, design studies that collect the best data for assessing outcomes and their determinants.

## Background

The challenges of implementing evidence-based innovations (EBIs) are widely recognized among practitioners and researchers. Context, broadly defined as everything outside the EBI [1], includes the dynamic and diverse array of forces working for or against implementation efforts [2]. As a result, implementation scientists have prioritized developing methods to understand and measure facets of context, which is necessary for all projects that involve planning, executing, or evaluating implementation efforts [3].

Theories that guide conceptualization of context abound and are often encapsulated within determinant frameworks [4, 5]; these frameworks delineate determinants (i.e., barriers or facilitators) that influence the outcome of implementation efforts. Knowledge of contextual barriers and facilitators is used to adapt EBIs [6], select and tailor implementation strategies [3, 7], and predict and/or explain implementation outcomes [8, 9]. Ultimately, the goal of this work is to increase knowledge about what works where and why to accelerate sustained integration of EBIs into routine practice.

The Consolidated Framework for Implementation Research (CFIR) is one of the most widely used frameworks within and outside implementation science [8, 10]. The original 2009 article invited critique in recognition of the need for the framework to evolve [2]. As implementation science has matured, gaps in the CFIR have been identified and updates are needed. Our team is developing the CFIR 2.0 based on a literature review and follow-up survey with authors. We encountered many recommendations in both the literature review and survey responses to add outcomes.

Although the CFIR is a determinant framework, users must develop, explore, and test theories of change that link determinants to implementation outcomes [8]. For example, Damschroder et al. identified seven CFIR determinants that were correlated with implementation outcomes using a mixed methods approach [9]; other regression- or Boolean-based analyses can be used to identify subsets of determinants that drive implementation outcomes [11]. In our trainings and consultations with new CFIR users, we have found that additional clarification and guidance is needed about which outcomes CFIR determinants influence and how to delineate determinants versus outcomes during coding and analysis. Currently published frameworks that define outcomes can be complicated to apply. For example, the Reach, Effectiveness, Adoption, Implementation, and Maintenance (RE-AIM) framework defines Maintenance outcomes at both the setting- and individual-level. As a result, users must be careful to delineate these levels because the determinants influencing Maintenance are different depending on how it is defined [12]. Furthermore, definitions are inconsistent across sources. For example, the RE-AIM framework defines Adoption as “the absolute number, proportion, and representativeness of: a) settings; and b) intervention agents (people who deliver the program) who are willing to initiate a program” [12]. Proctor et al.’s Implementation Outcomes Framework (IOF) defines Adoption as “the intention, initial decision, or action to try or employ an innovation” [13]. CFIR users will benefit from more clarity about (1) types of implementation outcomes, (2) implementation vs. innovation outcomes, and (3) determinants of implementation outcomes versus determinants of innovation outcomes.

We propose an Outcomes Addendum to the CFIR to address these issues. Our goal is not to create a new framework, but to help implementation researchers articulate which outcomes their studies are proposing to address, carefully consider the determinants that can affect those outcomes, and in turn, design studies that can collect the best data for measuring both outcomes and their determinants. The aim of this debate article is to describe the rationale for and conceptualization of the CFIR Outcomes Addendum, which draws on findings from the literature review and survey we conducted as part of our work on the CFIR 2.0 as well as the RE-AIM framework and the IOF [12, 13].

## Methods

We completed a literature review to identify recommendations from the published literature. More details will be provided in the future CFIR 2.0 manuscript. Briefly, we searched SCOPUS and Web of Science from 2009 (the year the CFIR was published) to July 6, 2020; we included all articles that mentioned the CFIR in the title and/or abstract. We identified 376 articles total; 16 articles included recommendations related to adding outcomes.

In addition to completing the literature review, we surveyed corresponding authors of included articles; there were 337 unique corresponding authors, but only 334 with contact information. Of the 334 contacted authors, 157 (47%) responded. The survey asked for recommendations to improve the CFIR including adding, removing, or modifying constructs and/or domains. Thirteen respondents recommended adding outcomes and three additional recommendations were related to outcomes, though they were not explicitly identified as such. The VA Ann Arbor Healthcare System IRB declared this study exempt from the requirements of 38 CFR 16 based on category 2.

## Proposed CFIR Outcomes Addendum

### Overview

There were recommendations to add both *implementation* and *innovation* outcomes to the CFIR from the literature review and survey. Hung et al. recommended inclusion of both types of outcomes because it would focus “the researcher’s attention squarely on the way that context shapes intermediate results and conditions, such as user acceptance, which in turn influence classic measures of an intervention's ultimate aims or outcomes” [14]. Some authors addressed this gap by linking the CFIR with another framework: nineteen used the RE-AIM framework and eight used the IOF [12, 13]. Other authors addressed this issue by adapting the CFIR to incorporate outcomes from both the RE-AIM framework and the IOF, including the CFIR-Process Redesign [14, 15] and the Care Transitions Framework [16].

The RE-AIM framework and the IOF were used to help inform broad categories of implementation outcomes and innovation outcomes included in the CFIR Outcomes Addendum. In addition, within implementation outcomes, we draw a distinction between anticipated implementation outcomes and actual implementation outcomes. Finally, we highlight contextual determinants (as described by CFIR constructs) as potential moderators of implementation outcomes versus innovation determinants (outside the scope of the CFIR) as potential moderators of innovation outcomes. Our goal is not to develop a new framework, but rather to clarify relationships between determinants and outcomes and to provide broad definitions that users can apply while using other frameworks.

### Implementation outcomes

The CFIR Outcomes Addendum broadly conceptualizes implementation outcomes as the success or failure of implementation. Anticipated implementation outcomes are based on perceptions or measures of the likelihood of future implementation success or failure, i.e., implementation outcomes that have not yet occurred. These outcomes are forward-looking; constellations of CFIR determinants across domains *predict* these outcomes. The concept of anticipated outcomes is well-established within the Sociology of Science and Technology field. Borup et al. assert that “[…] expectations can be seen to be fundamentally ‘generative,’ they guide activities, provide structure and legitimation, attract interest and foster investment. They give definition to role, clarify duties, offer some shape of what to expect and how to prepare for opportunities and risks” [17]. The terms anticipated and expected are used interchangeably and we chose the former term.

Actual implementation outcomes are based on perceptions or measures of current (or past) implementation success or failure, i.e., implementation outcomes that have occurred. These outcomes are backward-looking; constellations of CFIR determinants across domains *explain* these outcomes. Both anticipated and actual implementation outcomes can be assessed quantitatively or qualitatively.

Anticipated and Actual Implementation Outcomes include three broadly conceptualized outcomes based on our own work and the RE-AIM framework. While implementation research has tended to focus on initial implementation success, the importance of shifting from near-term implementation goals to long-term sustainment is increasingly clear. Several recommendations from both our literature review and survey responses highlighted the importance of capturing concepts of implementability and implementation, while even more discussed the importance of assessing Sustainability and Sustainment [14, 15, 18–26]. One survey respondent explained, “We added sustainability [sustainment] to the framework in our study. Planning for sustainability [sustainment] should begin at the earliest stages of the implementation process.” In a critique by Ilot et al. they recognized that when EBIs are not sustained, the result is a “waste of time, financial resources and leadership effort at a time of economic austerity” [18]. As a result, anticipated outcomes include adoptability, implementability, and sustainability, while actual outcomes include adoption, implementation, and sustainment. These major categories of implementation outcomes focus on the ultimate goals of implementation efforts: first, whether the decision is made to deliver the innovation (adoption); second, whether delivery of the innovation occurs (implementation); and third, whether the delivery of the innovation continues in the long-term. Table 1 lists definitions for each implementation outcome.

**Table 1:** Implementation outcomes definitions

	Anticipated outcomes	Actual outcomes
**Representativeness**	**Adoptability**: The likelihood key decision-makers will decide to put the innovation in place/innovation deliverers will decide to deliver the innovation.	**Adoption**: The extent key decision-makers decide to put the innovation in place/innovation deliverers decide to deliver the innovation.
	**Implementability**: The likelihood the innovation will be put in place or delivered.	**Implementation:** The extent the innovation is in place or being delivered.
	**Sustainability**: The likelihood the innovation will be put in place or delivered over the long-term.	**Sustainment:** The extent the innovation is in place or being delivered over the long-term.

**Fig. 1:**
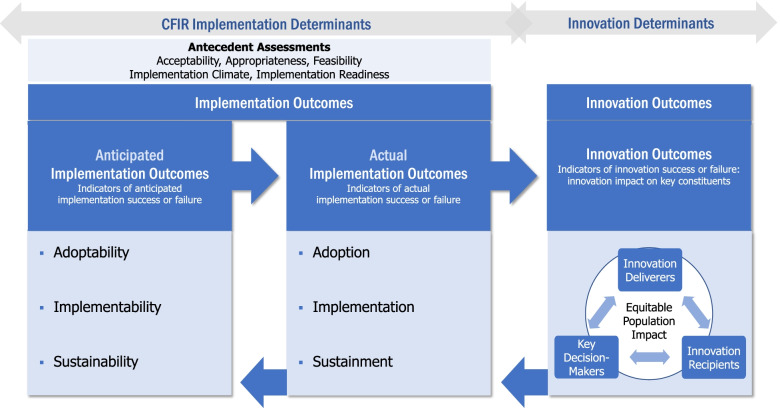
CFIR Outcomes Addendum diagram

As a result, although the IOF lists acceptability, appropriateness, and feasibility as implementation outcomes, these are not included as implementation outcomes in the CFIR Outcomes Addendum. These measures can be used to *predict* any anticipated or actual implementation outcome; for example, Weiner et al. developed measures for acceptability, appropriateness, and feasibility of an innovation and highlighted their role as potential predictors of adoption or implementation [27]. Thus, like Reilly et al., we classify these measures as “Antecedent Assessments” [28]. Additionally, the CFIR lists implementation climate and implementation readiness as higher-order constructs within the framework—each comprising multiple determinants. Since publication of the CFIR, there has been continued conceptual and measurement development for these concepts as potential predictors of implementation outcomes, but there is little consensus on their role within implementation theories [29–31]. Thus, we also place implementation readiness and implementation climate into the antecedent assessment category, which lies between CFIR determinants and implementation outcomes in Fig. 1. See Table 2 for a full mapping of RE-AIM Framework and IOF outcomes to the CFIR Outcomes Addendum. Given our goal to provide broad conceptualization of outcomes, many of the specific outcomes in existing frameworks map to broader concepts in the CFIR Outcomes Addendum.

**Table 2:** RE-AIM Framework and IOF outcomes mapped to the CFIR Outcomes Addendum

RE-AIM Framework [12] (except where noted)	Implementation Outcomes Framework [13]	CFIR Outcomes Addendum
*N/A: Not explicitly included*	**Acceptability:** The extent to which an innovation is perceived as “agreeable, palatable, or satisfactory.”	**Antecedent assessments** [28]
	**Appropriateness:** The “perceived fit, relevance, or compatibility of the innovation […] for a given practice setting, provider, or consumer; and/or perceived fit of the innovation to address a particular issue or problem.”	
	**Feasibility:** The extent to which an innovation “can be successfully used or carried out within a given agency or setting.”	
**Adoption:** “The absolute number, proportion, and representativeness of: a) settings; and b) intervention agents (people who deliver the program) who are willing to initiate a program.”	**Adoption:** “The intention, initial decision, or action to try or employ an innovation.”	**Actual Implementation Outcomes:** Adoption
^a^**Implementation (setting-level):** “The intervention agents’ fidelity to the various elements of an intervention’s protocol, including consistency of delivery as intended and the time required. Also includes adaptations made and the costs of implementation.	^b^**Penetration (setting-level):** “The integration of a practice within a service setting and its subsystems” which “can be calculated in terms of the number of providers who deliver a given service or treatment, divided by the total number of providers trained in or expected to deliver the service.”	**Actual Implementation Outcomes:** Implementation
**Fidelity:** “The extent to which the program is implemented consistently across different settings, staff, and patients.”	**Fidelity:** “The degree to which an intervention was implemented as it was prescribed in the original protocol or as it was intended by the program developer.”	
**Cost:** Costs of “replicating a program or policy in different settings;” “costs at the patient-, staff-, clinic-, and organizational levels;” “costs to deliver programs.”	**Cost:** “The cost impact of an implementation effort,” based on “the costs of the particular intervention, the implementation strategy used, and the location of service delivery.”	*N/A: Depending on how cost is defined, it may represent an implementation or innovation determinant or outcome.*
**Adaptation:** Adaptations made “prior to, during, and after program implementation.”	*N/A: Not explicitly included*	*N/A: Adaption is conceptualized as an implementation determinant in the CFIR Process Domain.*
^a^**Maintenance (setting-level):** The extent to which “a program or policy becomes institutionalized or part of the routine organizational practices and policies. Includes proportion and representativeness of settings that continue the intervention and reasons for maintenance, discontinuance or adaptation.”	^c^**Sustainability:** “The extent to which a newly implemented treatment is maintained or institutionalized within a service setting’s ongoing, stable operations.”	**Actual Implementation Outcomes:** Sustainment
**Setting Impact (setting-level):** Adoption X Implementation [28, 32]	*N/A: Not explicitly included*	*N/A: Not explicitly included*
**Reach (recipient-level): “**The absolute number, proportion, and representativeness of individuals who are willing to participate in a given initiative, intervention, or program.”	^b^**Penetration (recipient-level): **“The number of eligible persons who use a service, divided by the total number of persons eligible for the services.”	**Innovation Outcomes:** Innovation Impact on Recipients, Deliverers, and Key Decision-Makers
^a^**Implementation (recipient-level)** “Clients’ use of the intervention and implementation strategies.”		
**Effectiveness:** “The impact of an intervention on important outcomes, including potential negative effects, quality of life, and economic outcomes.”	^e^**Client Outcomes:** “Satisfaction, Function, and Symptomology.”	
^a^**Maintenance (recipient-level):** The extent to which “behavior is sustained 6 months or more after treatment or intervention.”		
^d^**Recipient Impact (recipient-level):** Reach X Effectiveness [28, 32]	*N/A: Not explicitly included*	
*N/A: Not explicitly included*	^e^**Service Outcomes: **“The extent to which services are safe, effective, patient-centered, timely, efficient, and equitable.”	

^a^Implementation and Maintenance: The RE-AIM framework includes definitions for Implementation and Maintenance at (1) the setting-level, which map to our *Implementation Outcomes* and at (2) the innovation recipient-level, which map to our *Innovation Outcomes*

^b^Penetration: The IOF provides a definition for Penetration at (1) the deliverer-level, which maps to our *Implementation Outcomes* and at (2) the recipient-level, which maps to our *Innovation Outcomes*

^c^Sustainability: Though the IOF uses the word Sustainability, the definition of this outcome maps to Sustainment in the CFIR Outcomes Addendum

^d^The CFIR Outcomes Addendum conceptualizes Recipient Impact for all constituents, which can be measured via Reach × Effectiveness [32] and Reach × Maintenance together [32–34]

^e^The CFIR Outcomes Addendum conceptualizes Client and Service Outcomes as potentially relevant to all constituents, e.g., patient-centeredness may be a priority for innovation deliverers and recipients, satisfaction may be a priority for key decision-makers and deliverers

A note on terminology: the terms Sustainability and Sustainment are commonly used colloquially and there is an entire “science of sustainability” that has much to offer to the “science of implementation” [17]. As a result, we chose these terms over Maintenance from the RE-AIM Framework.

### Innovation outcomes

The CFIR Outcomes Addendum broadly conceptualizes innovation outcomes as the success or failure of the innovation, based on the impact of the innovation on three important constituents: innovation recipients, innovation deliverers, and key decision-makers.

- Recipients are the human-beings for whom the innovation is designed to benefit, e.g., patients receiving treatment, students receiving a learning activity, or citizens receiving a city service.
- Deliverers are the human-beings who are directly or indirectly involved with delivering the innovation to recipients, e.g., clinicians delivering treatment to patients, teachers delivering a learning activity to students, or city employees delivering a city service to citizens.
- Key decision-makers are the human-beings who have authority within the implementing setting, whether it is a formal system or broader community, e.g., a hospital director deciding what treatment to deliver, a school superintendent deciding what learning activity to deliver, or a city mayor deciding what city service to deliver.

It is important to note that types of recipients and types of deliverers may overlap, e.g., when implementing a vaccination program for hospital employees, all employees are potential recipients while the specific employees delivering the vaccine (e.g., nurses who work within Employee Health) are also deliverers. These broad constituencies are based on feedback from CFIR users, who use the CFIR to plan and evaluate implementation of diverse innovations, both within and outside of healthcare.

While the outcomes important to innovation recipients (e.g., patients) and key decision-makers (e.g., hospital directors) are frequently prioritized in other frameworks (e.g., as reflected by the list of Client and Service Outcomes within the IOF), outcomes important to innovation deliverers (e.g., clinicians) are often not prioritized. Consideration of clinicians (and other employees) motivated evolution of the “Triple Aim” (enhancing patient experience, improving population health, and reducing costs) [35] to the “Quadruple Aim,” which added an aim of improving the work-life and well-being of clinicians and staff [36]. Ideally, implementation of innovations will produce benefit for not only innovation recipients and key decision-makers, but also innovation deliverers, e.g., reducing burnout, improving work experience.

Sustainment of outcomes may be strengthened when goals are aligned between these three key constituencies, each of whom are likely to have different priorities and interests [37–40]. For example, an innovation that improves patient function (an important outcome to patient recipients) is unlikely to be sustained if it increases burnout for clinicians (an important outcome to clinician deliverers) and/or increases system costs (an important outcome to key decision-makers). Because CFIR users are focused on achieving and sustaining implementation, it is important to consider which outcomes are most important to which people. We believe that by highlighting the *human-beings* impacted by Innovation Outcomes, the CFIR Outcomes Addendum will help researchers and organizations orient to values of humanism and equity. Figure 1 illustrates the components of the CFIR Outcomes Addendum.

In Fig. 1, right facing arrows at the top of the figure illustrate the temporal nature of (1) anticipated and actual implementation outcomes and (2) implementation outcomes and innovation outcomes. The right facing arrow between anticipated and actual implementation outcomes illustrates the generative nature of anticipated outcomes (see the “Implementation outcomes” section above). The right facing arrow between implementation and innovation outcomes illustrates the foundational premise within implementation science that successful implementation is a necessary pre-condition to achieving maximum innovation benefits [41]. For example, an effective innovation will fail to produce expected outcomes if it is poorly implemented; this may result in a “Type III” error, when evaluators conclude that the innovation is ineffective, when in fact that same innovation may have met or exceeded expectations if it had been properly implemented [42]. In addition, left facing arrows across the bottom of the figure illustrate the reinforcing loop that can emerge when the positive impact of an innovation inspires continued commitment to implementation and sustainment [43].

A note on terminology: We have opted to use the term Innovation to be broadly inclusive of other terms. Rogers’ classic Diffusion of Innovation theory defines innovation as an idea, practice, or object that is perceived as new by an individual or other unit of adoption. If an idea seems new within a setting or for an individual, it is an innovation [44]. This is a broad definition and includes any “thing” that is being implemented [45]: Innovations can include, e.g., medications, medical devices, behavior change interventions, technology, and more—or any combination. An innovation is ideally supported by a “strong evidence-base” before it is implemented. However, we also recognize there is lack of agreement on what types of evidence warrant implementation [46–48] and there is a compelling need to dismantle knowledge-building silos (e.g., clinical trialists versus implementation scientists) to translate innovations more quickly into practice [49]. Thus, we chose the term “innovation” to acknowledge that implementation can occur with innovations that are supported by diverse sources and types of evidence.

## CFIR implementation determinants vs. innovation determinants

When collecting data, researchers must be clear about the goal of data collection: (1) to predict and/or explain implementation outcomes based on implementation determinants (this is within the scope of the CFIR) or (2) to predict and/or explain innovation outcomes based on innovation determinants (this is outside the scope of the CFIR). The following section explores the roles of implementation versus innovation determinants.

### Implementation determinants

CFIR implementation determinants capture setting-level barriers and facilitators that predict and/or explain *antecedent assessments* and/or *anticipated or actual implementation outcomes.* These determinants are denoted by the gray arrow in Fig. 1 labeled *CFIR implementation determinants.* Data (qualitative and/or quantitative) on these determinants is best collected from individuals who have influence and/or authority related to implementation (usually within the implementing setting); these typically include the key decision-makers and individuals implementing and/or delivering the innovation.

Although over 20 users recommended adding a domain and/or constructs to collect data directly from recipients, the CFIR is not the appropriate framework to use for this purpose unless recipients are also helping to implement and/or deliver the innovation. As reflected by Orlando et al., it is disappointing to note that “… while patients are part of the health-care organization and are essential to assessing *intervention [innovation]* effectiveness, they are a less influential component of *implementation* success in health-care settings than administrators and physicians” (emphases added) [50]. Although hospital systems are increasingly prioritizing patient-centered care, convening patient advisory boards, and involving patients in co-design of initiatives [51, 52], these efforts have not yet resulted in true power-sharing between innovation recipients and key decision-makers [53].

As a result, direct data collection from recipients does not usually inform implementation outcomes. Instead, data collection from key decision-makers and individuals implementing and/or delivering the innovation about *their* perceptions of recipients (e.g., recipient characteristics and needs), and how those perceptions encourage (or discourage) completing implementation, informs Implementation Outcomes. Although the CFIR is often not appropriate for use with recipients (because they rarely hold roles as key decision-makers or innovation implementers/deliverers), we hope that will change. Recipients *should* have greater influence, authority, and power in healthcare systems; the CFIR 2.0 will highlight the importance of implementation teams *including* innovation recipients (and innovation deliverers) as members. When recipients serve in that role, we strongly encourage using the CFIR to collect data about implementation determinants from them—because they are *also* implementation team members. Ultimately, equitable population impact is only possible when recipients are integrally involved in implementation and all key constituencies share power and make decisions together.

### Innovation determinants

Innovation determinants capture recipient-level characteristics and/or experiences with the innovation that predict and/or explain *innovation outcomes.* These determinants are denoted by the gray arrow in Fig. 1 labeled *Innovation determinants.* Data (qualitative and/or quantitative) on these determinants is best collected from recipients. Innovation determinants include constructs or measures that are based on the theoretical framework underlying the innovation. For example, in a “small change” weight loss intervention designed for patients, innovation determinants included patient-level demographics, motivation and intention, and self-efficacy because the intervention was guided by social-psychological and goal-conflict theories [54]. This innovation was tested within a randomized clinical trial [55] and a subset of patient characteristics (innovation determinants) were explored in secondary analyses to help explain Innovation Outcomes [56–59]. The CFIR is not designed to capture these theory-derived determinants of Innovation Outcomes.

## Conclusion

As implementation science matures as a discipline, frameworks must mature too [60, 61]. In this debate article, we propose the inclusion of an Outcomes Addendum to the CFIR. Our goal is to offer clear conceptual distinctions between the types of outcomes for use with the CFIR, and perhaps other determinant implementation frameworks as well. These distinctions can help bring clarity as researchers consider which outcomes are most appropriate to evaluate in their research and to help center those outcomes on multiple key constituencies for sustained outcomes. We hope that sharing this in advance will generate feedback and debate about the merits of our proposed addendum.

## Data Availability

The datasets used and/or analyzed during the current study are available from the corresponding author on reasonable request.

## Abbreviations

CFIR: Consolidated Framework for Implementation Research
EBI: Evidence-based innovation
IOF: Implementation Outcomes Framework
RE-AIM Framework: Reach, Effectiveness, Adoption, Implementation, and Maintenance Framework
